# Association between emphysema and other pulmonary computed tomography patterns in primary varicella pneumonia: A retrospective cohort study

**DOI:** 10.1097/MD.0000000000038185

**Published:** 2024-05-17

**Authors:** Meng Li, Guijuan Zhu, Yajun Guo, Qing Ye

**Affiliations:** a Department of Pulmonary & Critical Care Medicine, Renmin Hospital, Hubei University of Medicine, Shiyan, Hubei, PR China; b Department of Gynecology, Renmin Hospital, Hubei University of Medicine, Shiyan, Hubei, PR China.

**Keywords:** chest CT, emphysema, lung, pulmonary infiltrates, varicella

## Abstract

This study aims to evaluate chest computed tomography (CT) findings in hospital patients with primary varicella pneumonia (PVP). We retrospectively analyzed CT images of 77 PVP patients using 3D Slicer, an open-source software, to model lesions and lungs. This retrospective cohort study was approved by the Institutional Review Board (Ethical Committee, Renmin Hospital, Hubei University of Medicine, Shiyan, China) and waived the requirement for written informed consent. The left lung was more frequently and severely affected in PVP, with significant differences between the 2 groups in CT involvement percentage of each lung region, except for total lung inflation. Group A showed higher median percentages of lung collapse compared to Group B. The extent of left lung involvement is a critical predictor of emphysema in PVP patients, highlighting the importance of also monitoring the right lung for more severe cases. Lower emphysema levels correspond to more collapsed and infiltrated lung segments, suggesting a more severe clinical presentation.

**Summary Box**

Varicella pneumonia usually presents with a primary varicella infection at first. The presenting characteristics are the main characters in diagnosing varicella pneumonia. Multiple lung nodules are the main character presented in varicella pneumonia on computed tomography. The percentage of affected left lung is an independent predictor of emphysema in PVP patients. In the future, we need to focus on the right lung of the patient as it is more affected. The people with lower levels of emphysema may have more collapsed and infiltrated segments. The more collapsed segments may lead to more serious clinical features.

## 1. Introduction

As a type of herpes virus, varicella-zoster virus (VZV) can cause herpes zoster/shingles (due to reactivation of latent infection) and varicella (primary infection).^[1]^ As a self-limited and benign illness,^[2]^ it sometimes can result in severe complications when the patients are in both immunocompetent and immunocompromised. Primary varicella pneumonia, a complication of herpes zoster,^[3]^ is a rare event. It can develop a secondary bacterial infection of the skin lesions and then pneumonia associated with VZV infection. The diagnosis of primary varicella pneumonia is mainly based on symptoms of cough and fever and chest CT findings. Varicella pneumonia can present as potentially life-threatening complications of varicella and is uncommon in patients. As a serious complication of chickenpox, primary varicella pneumonia is estimated to occur in 1 out of 400 infections.^[4]^

As a highly contagious disease, many body systems can be affected such as the lungs, skin, and central nervous system. Smoking can lead to decrease the activation of alveolar macrophages; it will increase the susceptibility to varicella pneumonia. As a complication from VZV,^[5]^ varicella pneumonia usually presents with a primary varicella infection at first,^[6]^ and then may develop tachypnoea, coach, fever, dyspnea, chest tightness, and even hemoptysis. The presenting characteristics is the main ones in diagnosing varicella pneumonia. Multiple lung nodules are the main character presented in varicella pneumonia on computed tomography (CT).^[7]^

As a common infection in childhood, varicella usually affects children aged 2 to 8 years. However, when it is found in adults, varicella pneumonia will be the most common and serious complication. According to the report, the incidence of varicella pneumonia in adults is 25-fold greater than in children. Patients in chronic lung disease and impaired immune status may have a higher percentage develop primary varicella pneumonia infection. We usually use antiviral therapy in treating varicella while it may not be useful for varicella pneumonia. Radiologists and radiology residents need to identify this type of pneumonia by characteristic features.^[8]^ Diffuse pulmonary nodules can be found in active varicella pneumonia. It will be presented as diffuse, calcified pulmonary micronodules in healed varicella pneumonia.^[9]^

In rare cases, PVP can be a fatal complication of VZV. Patients will be found with respiratory distress after the onset of a rash within 1 to 6 days.^[10]^ High-resolution CT can be able to more precisely identify varicella pneumonia compared with chest radiographs.^[11]^ The most significant presence of acute Varicella pneumonia is ground-glass opacities while this finding would not present in healed varicella pneumonia. Bronchoalveolar lavage,^[12]^ polymerase chain reaction (PCR) checks the presence of varicella-zoster viral DNA,^[13–16]^ can be utilized in diagnosis in varicella pneumonia. Skin biopsies can also be used in testing for VZV which charactered with subepidermal vesicles with multinucleated giant cells.^[17]^ There are various risk factors to develop varicella pneumonia such as chronic lung diseases, previous or current smokers, third trimester of pregnancy and severity of the skin rash.^[18]^

## 2. Materials and methods

This retrospective observational study was approved by the Institutional Review Board (Ethical Committee, Renmin Hospital, Hubei University of Medicine, Shiyan, China) and waived the requirement for written informed consent.

### 2.1. Study population and data collection

We conducted this retrospective cohort study in Renmin Hospital, Hubei University of Medicine, Shiyan between February 4 and April 25, 2022, with varicella infection included in our study. Widespread pleomorphic rash and fever are clinical character based on the diagnosis of varicella. The varicella pneumonias were all diagnosed by radiological findings which present new onset of respiratory symptoms within 10 days. If the patients were diagnosed to have chronic lung diseases such as allergic bronchopulmonary aspergillosis, bronchiectasis, and chronic obstructive pulmonary disease, they will be excluded.

### 2.2. CT image acquisition and analysis

CT scans of the chest are acquired simultaneously from the lung toward the apex of the lung. We use a 64-slice and a 16-slice scanner (LightSpeed16) to get it. The CT images were all taken by the 2 authors of this paper for acquisition. Images used standard lung window settings (width, 1200 HU; level, −500 HU) at slice thicknesses 0.9 to 1.5 mm and sharp kernels.

The open-source 3D Slicer, version 4.13.1 (https://www.slicer.org) was used to compare damaged lung volume. The software can identify 5 regions of interest: “Bulla/emphysema,” “Inflated,” “Infiltrated,” “Collapsed,” and “Lung Vessel.” The results are viewed in standard colors: “Bulla” = black, “Inflated” = blue, “Infiltrated” = yellow, “Collapsed” = pink and “Vessel” = red. It used 3D Slicer’s segment editor “Threshold” and “Grow from Seeds” functions to generate segments. The total results of the segmentation included: functional total lung volume (inflated, percentage of total lung volume), functional left lung volume (inflated, percentage of left lung volume), functional right lung volume (inflated, percentage of right lung volume), total lung volume (100%), left lung volume (percentage of total lung volume), right lung volume (percentage of total lung volume), affected total lung volume (infiltrated and collapsed total lung volume, percentage of total lung volume), affected left lung volume (infiltrated and collapsed left lung volume, percentage of left lung volume), affected right lung volume (infiltrated and collapsed right lung volume, percentage of right lung volume).

### 2.3. Divide into groups

According to the study of Castaldi, patients in %LAA-950 emphysema equal to or >10% the moderate centrilobular pattern would predominate. And the predominant lesion lung class was the mild centrilobular pattern in %LAA-950 emphysema of <10%. Therefore, we divided the 77 patients with PVP into the following 2 groups: (A) 24 individuals with %LAA-950 emphysema <10% and (B) 53 individuals with %LAA-950 emphysema greater than or equal to 10%.

### 2.4. Threshold-based emphysema measures

Each lung CT scan calculates the threshold-based %LAA-950 measure below the 2950 Hounsfield unit threshold separately.

### 2.5. Statistical analysis

Data analysis was carried out using SPSS (version 25.0).

We summarized continuous variables using median or mean ± SD and interquartile range (IQR) when appropriate. Categorical variables are all presented as n (%). The differences in demographic data were compared with Mann–Whitney *U* test, Chi-square, and Fischer exact tests using permutation method for multiple comparisons. Differences between collapsed, pulmonary infiltrates, affected, emphysema of patients with CT findings were assessed by Wilcoxon and Kruskal–Wallis test. In all statistical analyses, *P* value < .05 was considered statistically significant.

## 3. Results

### 3.1. Clinical findings

The clinical data of the 77 primary varicella pneumonia patients are shown in Table 1, of whom 53 were in Group A and 24 in Group B. Most patients were male (54.5%). The age of the patients ranged from 19 to 85 years, with a mean of 45.70 ± 14.91 years. The most common clinical symptoms were fever (60/77, 77.9%) and cough (26/77, 33.7%). Hypertension was the most common comorbidity of primary varicella pneumonia (9/77, 11.6%), followed by diabetes (4/77, 5.1%) and coronary artery disease (4/77, 5.1%). There were significant differences between the 2 groups in terms of cough (*P* = .016), myalgia (*P* = .013), and dyspnea (*P* = .033). Besides, we must note that the percentage of fever in group B is 83% which is so significance. Table 1 shows the detailed patient characteristics.

**Table 1:** Demographics, comorbidities, symptoms of patients.

Variables	Factor	All patients (n = 77)	A (n = 53)	B (n = 24)	*P* value
Age (yr), median (IQR)		45.70 ± 14.91	46.41 ± 14.63	42.50 ± 16.40	.541^b^
Gender, n (%)	Male	41 (54.5)	30 (55.6)	12 (50)	.123^a^
	Female	36 (45.5)	23 (44.4)	12 (50)	
Comorbidities n (%)	Diabetes	4 (5.1)	3 (5.6)	1 (8.3)	.333^a^
	Hypertension	9 (11.6)	6 (11.1)	3 (16.7)	.184^a^
	Coronary artery disease	4 (5.1)	3 (5.6)	1 (8.3)	.233^a^
	Chronic lung disease	0 (0)	0 (0)	0 (0)	–
	Chronic liver disease	0 (0)	0 (0)	0 (0)	–
	Chronic renal failure	0 (0)	0 (0)	0 (0)	–
	Malignancy	0 (0)	0 (0)	0 (0)	–
	postpartum	2 (3.0)	1 (1.9)	1 (4.3)	1.304^a^
Symptoms	Fever	60 (77.9)	40 (74.1)	20 (83)	3.919^a^
	Cough	26 (33.7)	19 (35.2)	7 (29.3)	.016^a^
	Dyspnea	17 (22.7)	12 (22.2)	5 (20.0)	.033^a^
	Myalgia	5 (7.6)	4 (7.4)	1 (4.3)	.013^a^

a Chi-square test.

b Fischer exact test.

### 3.2. Imaging findings

Patients with primary varicella pneumonia were all divided into 2 different groups with whether the %LAA-950 emphysema was >10% or not. In group A, it was <10% and in group B, it was greater than or equal to 10%. We analyzed the CT scans of these patients in both 2 groups. Segments were all created based on the Hounsfield units with a predefined threshold range.

Overall, the left lung (Group A: left lung 19.00%/right lung 17.50%, Group B: left lung 12.00%/right lung 10.50%) was the region of the lung most frequently involved by primary varicella pneumonia. In addition, for lung collapse, the left lung (group A: left lung 4.85%/right lung 4.65%, group B: left lung 3.75%/right lung 3.25%) was also more affected than the right lung. There were important differences between the 2 groups in the percentage of CT involvement of each one region (*P* < .05), except for the total lung which was involved in inflation (*P* = .162). In group A, the median percentage of collapsed left lung was 4.85 (4.18–6.93) for the right lung 4.65 (3.88–6.75), and for the total lung 4.65 (4.10–7.10). In contrast, the median percentage of collapsed left lung in group B was 3.75 (2.95–4.00), for the right lung 3.25 (2.83–3.78), and for the total lung 3.35 (2.93–3.93) (Table 2).

**Table 2:** Comparison of the percentage of involvement of each lung zone between the 2 groups.

Segment	Lung	A (%)	B (%)	*P* value
Inflated affected	Total	77.00 (69.25–84.00)	73.00 (72.00–74.00)	.162
	Left	82.00 (70.00–86.00)	87.00 (83.50–90.75)	<.001
	Right	82.50 (69.75–87.00)	86.50 (85.00–90.00)	<.001
Emphysema	Total	1.55 (0.30–2.85)	13.95 (10.43–17.90)	<.001
	Left	1.20 (0.40–3.73)	13.00 (11.53–18.73)	<.001
	Right	0.95 (0.20–2.50)	13.65 (10.18–16.83)	<.001
Infiltrated	Total	14.75 (9.53–22.40)	8.65 (7.10–11.35)	.001
	Left	14.65 (9.93–22.50)	9.30 (6.80–11.15)	.001
	Right	13.85 (9.30–22.33)	8.30 (7.33–11.48)	.003
Collapsed	Total	4.65 (4.10–7.10)	3.35 (2.93–3.93)	<.001
	Left	4.85 (4.18–6.93)	3.75 (2.95–4.00)	<.001
	Right	4.65 (3.88–6.75)	3.25 (2.83–3.78)	<.001
Affected	Total	18.00 (13.00–30.00)	11.50 (10.00–15.00)	.001
	Left	19.00 (14.00–30.00)	12.00 (10.00–14.75)	.001
	Right	17.50 (13.00–30.25)	10.50 (10.00–15.00)	.001

Median of the percentage, the confidence interval for the median.

In our univariate analysis, almost all factors but except cough, myalgia, dyspnea, and total affected lung inflation could influence %LAA-950 emphysema. In our multivariable analysis, otherwise, patients with primary varicella pneumonia who had a higher right lung infiltrated (odds ratio 1.12, 95% CI 0.01–10.22; *P* = .028) and affected (odds ratio 1.28, 95% CI 0.02–10.11; *P* = .037) were most likely to have >10% %LAA-950 emphysema (details in Table 3).

**Table 3:** Univariable and multivariable analysis and their associations.

Variable	Lung	Univariate analysis	95% CI	*P* value	Multivariate analysis	95% CI	*P* value
		OR			OR		
Cough		0.92	0.24 to 3.46	.901	–	–	–
Dyspnea		1.17	0.27 to 5.00	.841	–	–	–
Myalgia		1.14	0.12 to 11.18	.912	–	–	–
Inflated affected	Total	1.00	0.95 to 1.05	.841	–	–	–
	Left	1.38	1.08 to 1.77	.010	1.14	0.52 to 2.51	.744
	Right	1.32	1.05 to 1.65	.015	1.24	0.08 to 18.27	.878
Infiltrated	Total	0.75	0.58 to 0.97	.025	–	–	.455
	Left	0.73	0.56 to 0.96	.022	–	–	.505
	Right	0.79	0.63 to 0.98	.029	1.12	0.01 to 10.22	**.028**
Collapsed	Total	0.05	0.01 to 0.33	.002	–	–	.706
	Left	0.06	0.01 to 0.38	.003	5.02	–	.879
	Right	0.08	0.02 to 0.38	.002	0.47	–	.947
Affected	Total	0.76	0.60 to 0.96	.019	16.9	–	.088
	Left	0.71	0.54 to 0.93	.014	1.18	0.01 to 10.27	.049
	Right	0.78	0.63 to 0.96	.018	1.28	0.02 to 10.11	**.037**

### 3.3. CT image comparison

**Figure 1.**
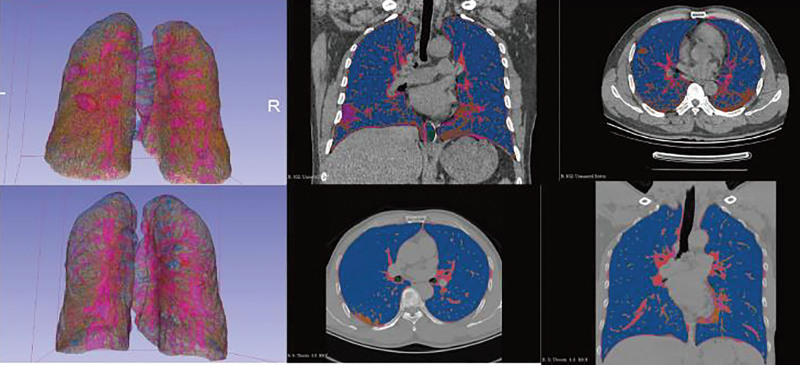
A compared image from 2 groups.

The axial chest CT scans of 56-year-old women from Group A and 55-year-old women from Group B were compared side by side in 3D Slicer (Fig. 1).

## 4. Discussion

In our study, 77 patients with primary varicella pneumonia were evaluated. All 77 patients were admitted from the Renmin Hospital, Hubei University of Medicine, China from February 4, 2022, to April 25, 2022. We divided patients in 2 groups by a threshold of %LAA-950 emphysema of 10%. We used 3D Slicer to analyze the percentage of involvement from each lung region between the 2 groups A and B. We found cough, myalgia, and dyspnea those common clinical symptoms affected both groups of patients. The right lung was the most frequently involved site in primary varicella pneumonia. We used an in-depth analysis of the percentage of patients that were involved in each lung zone in 2 groups. Additionally, inflated affected left lung and right lung, infiltrated left lung and right lung and total lung, emphysema left lung and right lung and total lung, affected left lung and right lung and total lung, collapsed left lung and right lung and total lung, all are differed importantly between the 2 groups. We have proved that a greater proportion of the right lung was affected in primary varicella pneumonia patients in univariate and multivariate analysis, most likely resulting in %LAA-950 emphysema can >10%.^[14]^

In our study, the Lung CT Analyzer was used to identify 5 regions of interest. There are significant differences between our 2 groups in all regions from our study.^[19]^ In the past, there is little study about primary varicella pneumonia.^[20]^ It is very important for patients to know if the primary varicella pneumonia is series or not. Even now, few studies pay attention to the relationship between lung CT and the clinical features of patients with primary varicella pneumonia.^[21]^ We need to recognize which may have been neglected and which patients may be serious through the lung CT. The collapsed and infiltrated segments there are, the more likely they will lead to the more serious clinical features.^[22]^ However, earlier CT expression can play a significant role in helping doctors to manage patients in primary varicella pneumonia.^[23]^

In the introduction, we know that CT of the chest plays a very vital role in patients with primary varicella pneumonia.^[24]^ Sometimes severe varicella pneumonia is an acute respiratory illness in adults, it may require mechanical ventilation. It is very necessary for doctors to analyze the CT of the chest when the primary varicella pneumonia has not been found.^[23]^

There are also some limitations to our study. Our retrospective study is a short time, during the period the results may be influenced.^[25]^ The serological of varicella had not been used in our study. Due to the limited number of patients, the result may not be enough trusted.^[26]^ In the future, we need more clinical and laboratory factors of varicella. Nowadays, computer vision-based detection algorithms for the detection of patients in the world still receive less attention. We hope more and more doctors can pay attention to the new method in clinic. A larger sample size and the baseline CT examination would be required to describe the full population of primary varicella pneumonia patients.^[27]^

## 5. Conclusion

There have been many computer vision and artificial intelligence methods for disease control and management. It can extract features from radiological images. However, these methods have not been cited in primary varicella pneumonia.^[28]^ Through our retrospective study, the 2 groups have significant differences in patients. Attention needs to be paid to the patient’s right lung, as it was more likely affected. Moreover, the collapsed and infiltrated segments of the right lung must be more significant in the treatment of primary varicella pneumonia. In conclusion, even though primary varicella pneumonia is a rare complication, it is still very serious. Our report emphasizes how critical timely diagnosis through lung CT can help primary varicella pneumonia patients find danger in lung earlier.^[29]^

## Acknowledgments

We apologize to the many authors whose studies are important but could not be cited due to space limitations.

## Author contributions

**Data curation:** Meng Li.

**Formal analysis:** Guijuan Zhu, Yajun Guo.

**Conceptualization:** Qing Ye.
